# Identification of agonists for a group of human odorant receptors

**DOI:** 10.3389/fphar.2015.00035

**Published:** 2015-03-03

**Authors:** Daniela C. Gonzalez-Kristeller, João B. P. do Nascimento, Pedro A. F. Galante, Bettina Malnic

**Affiliations:** ^1^Department of Biochemistry, Institute of Chemistry, University of São PauloSão Paulo, Brazil; ^2^Centro de Oncologia Molecular, Hospital Sírio-LibanêsSão Paulo, Brazil

**Keywords:** human odorant receptor, human olfactory receptor, odorants, heterologous expression, functional screening, orphan receptors, GPCRs, odorant perception

## Abstract

Olfaction plays a critical role in several aspects of the human life. Odorants are detected by hundreds of odorant receptors (ORs) which belong to the superfamily of G protein-coupled receptors. These receptors are expressed in the olfactory sensory neurons of the nose. The information provided by the activation of different combinations of ORs in the nose is transmitted to the brain, leading to odorant perception and emotional and behavioral responses. There are ~400 intact human ORs, and to date only a small percentage of these receptors (~10%) have known agonists. The determination of the specificity of the human ORs will contribute to a better understanding of how odorants are discriminated by the olfactory system. In this work, we aimed to identify human specific ORs, that is, ORs that are present in humans but absent from other species, and their corresponding agonists. To do this, we first selected 22 OR gene sequences from the human genome with no counterparts in the mouse, rat or dog genomes. Then we used a heterologous expression system to screen a subset of these human ORs against a panel of odorants of biological relevance, including foodborne aroma volatiles. We found that different types of odorants are able to activate some of these previously uncharacterized human ORs.

## Introduction

Humans can discriminate a vast number of odorants with diverse chemical structures (Bushdid et al., 2014). Odorants are detected by a large family of odorant receptors (ORs) expressed in the cilia of olfactory sensory neurons located in the nose (Buck and Axel, 1991). The ORs belong to the super-family of G-protein coupled receptors (GPCRs) and are extremely diverse in their amino acid sequences, consistent with the ability to recognize a large variety of odorants (Buck and Axel, 1991; Malnic et al., 2004). When activated by particular odorants, these GPCRs couple to an olfactory specific G protein, denominated Gαolf (Jones and Reed, 1989), leading to the activation of adenylyl cyclase III, increase in cAMP concentration, activation of cyclic nucleotide gated channels (CNGC2) and olfactory neuron depolarization (Mombaerts, 2004; Pifferi et al., 2010). The information is then passed on to different regions of the brain leading to odorant perception and emotional and behavioral responses (Buck, 1996).

Humans have a large number of OR genes (~400 genes) (Malnic et al., 2004), most of which have been shown to be expressed in the human olfactory epithelium (Verbeurgt et al., 2014). Odorant perception is initiated through the activation of specific combinations of ORs by a given odorant (Malnic et al., 1999). Identification of the odorant specificities of the ORs should therefore provide information about how odorant identities are encoded in the olfactory system. Efficient expression of ORs in heterologous systems is a necessary step to determine their odorant preferences. However, heterologous expression of ORs is usually poor and odorant specificities remain unknown for the majority of the human ORs.

Different strategies have been used to improve functional analysis of this type of GPCRs (Malnic, 2007; Peterlin et al., 2014). For example, fusion of the 20 N-terminal amino acids of rhodopsin (Rho tag) to the N-terminal region of ORs facilitates cell surface expression (Krautwurst et al., 1998; Wetzel et al., 1999). It has also been shown that coexpression with the olfactory specific protein RTP1-S (receptor transporting protein 1, short version) promotes OR surface expression in HEK293T cells (Saito et al., 2004; Zhuang and Matsunami, 2007). In addition, coexpression with the guanine nucleotide exchange factor Ric-8B, which amplifies receptor signaling through Gαolf *in vitro*, also enhances functional expression of ORs in heterologous cells (Von Dannecker et al., 2005, 2006). These and other approaches have been used to deorphanize ORs during the last years, so that to date around 40 human ORs have been linked to odorants (Wetzel et al., 1999; Matarazzo et al., 2005; Sanz et al., 2005; Jaquier et al., 2006; Neuhaus et al., 2006; Schmiedeberg et al., 2007; Saito et al., 2009; Jaeger et al., 2013; Busse et al., 2014; Mainland et al., 2014; McClintock et al., 2014; Shirasu et al., 2014).

In this work, we aimed to identify agonists for a group of selected human ORs, which may be particularly relevant to the human species. We first searched for ORs that are present in the human genome but absent from the genomes of other mammalian species, such as mouse, rat and dog. By using this approach, we selected 22 ORs, the majority of which are orphan receptors, with unknown agonists. Then, we used a heterologous functional assay to screen these receptors against a group of biologically relevant odorants. Odorants able to activate some of the selected human ORs were identified.

## Materials and methods

### Selection of human OR genes

The genome sequence from *Mus musculus* (version mm9), *Rattus norvegicus* (version rn4), *Canis familiaris* (version canFam2) and *Pan troglodytes* (version panTro2) were downloaded from UCSC Genome Browser (http://genome.ucsc.edu). All human genes were downloaded from Entrez GENE (Maglott et al., 2007), and the genes annotated as “olfactory receptor” were selected. These sequences were also manually inspected to confirm their annotation as ORs. Sequences were translated and ORs without a coding region or with a coding region smaller than 900 nucleotides were classified as pseudogenes; ORs with coding regions greater than 900 nucleotides were classified as functional ORs. A list of 403 functional ORs was obtained. The functional ORs were aligned against the mouse, rat, dog and chimpanzee genomes using the BLAT program (default parameters) (Kent, 2002).

### Analysis of amino acid sequences

The amino acid sequences corresponding to the complete coding regions of the nine human ORs analyzed in the functional assays were aligned using multiple sequence alignment by ClustalW (http://www.genome.jp/tools/clustalw/) (Supporting Material). The phylogenetic tree in **Figure 4** was prepared based on the amino acid sequences of the 22 selected human ORs by using the PhyML 3.0 software (Guindon et al., 2010) and the bootstrapping procedure for branch support values (Phylogeny.fr, http://phylogeny.lirmm.fr/phylo_cgi/index.cgi).

### Cloning of the human ORs

The full length DNA sequences from the human ORs were amplified from human genomic DNA by PCR and subcloned into the pcDNA3.1(-) expression vector (Invitrogen). The OR sequences were also cloned with the 5′ addition of a Rho tag DNA sequence, as previously described (Von Dannecker et al., 2006). The sequences of the cloned ORs were checked by DNA sequencing on an ABI PRISM 3100 Genetic Analyzer (Hitachi, Japan). Primer sequences used for PCR amplification and cloning are:

**Table:** 

OR2T34f	5′ CCGCTCGAGATGTGCTCAGGGAATCAGAC 3′
OR2T34r	5′ GGGGTACCCTACTTTTCTTGGTTCATTCTTG 3′
OR1G1f	5′ CCGCTCGAGATGGAGGGGAAAAATCTGAC 3′
OR1G1r	5′ GGGGTACCCTAAGGGGAATGAATTTTCCG 3′
OR5AC2f	5′ CCGCTCGAGATGGATATATCAGAGGGAAATAAG 3′
OR5AC2r	5′ GGGGTACCTTACTTCCTTATAACTCTTCTCAATG 3′
OR1L3f	5′ CCGCTCGAGATGGGAATGTCCAACCTGAC 3′
OR1L3r	5′ GGGGTACCTCAGGGTCCACAGATTTTATTG 3′
OR2B3f	5′ CCGCTCGAGATGAATTGGGAAAATGAGAGCTC 3′
OR2B3r	5′ GGGGTACCATCACAATGGAGTACTTCTTATTTC 3′
OR2G2f	5′ CCGCTCGAGATGGGGATGGTGAGACATAC 3′
OR2G2r	5′ GGGGTACCTCATAAAATATTTACTCCCAGAGC 3′
OR2M4f	5′ CCGCTCGAGATGGTGTGGGAAAACCAGAC 3′
OR2M4r	5′ GGGGTACCTCATATTAACTTTCTTTTCTTCAG 3′
OR5B17f	5′ CCGCTCGAGATGGAGAATAATACAGAGGTGAG 3′
OR5B17r	5′ GGGGTACCTTAAAAGACTGAATCTAGAGAATATTTTG 3′
OR2T10f	5′ CCGCTCGAGATGCGGCTGGCCAACCAGAC 3′
OR2T10r	5′ GGGGTACCTTAATATGGAGGTTTCTGCACGC 3′

### Odorants

The panel of odorants was provided by Givaudan do Brasil Ltda and contained the following odorants (the IUPAC nomenclature is given in parenthesis): maltyl isobutyrate ((2-trimethyl-4-oxopyran-3-yl) 2-methylpropanoate); fructone (ethyl 2-(2-trimethyl-1,3-dioxolan-2-yl)acetate); eugenyl acetate ((2-methoxy-4-prop-2-enylphenyl) acetate); terpinyl acetate (2-(4-trimethyl-1-cyclohex-3-enyl)propan-2-yl acetate); benzyl acetate; bornyl acetate ((1,7,7-trimethyl-6-bicyclo[2.2.1]heptanyl) acetate); manzanate (ethyl 2-methylpentanoate); hedione (methyl 3-oxo-2-pentylcyclopentaneacetate); jasmopyrane ((3-pentyloxan-4-yl) acetate); allyl amyl glycolate ((prop-2-enyl 2-(3-methylbutoxy)acetate); linalyl acetate (3,7-dimethylocta-1,6-dien-3-yl acetate); scentenal (octahydro-5-methoxy-4,7-methano-1H-indene-2-carboxaldehyde); cinnamaldehyde (3-phenylprop-2-enal); benzaldehyde (methyl benzoate); citronellal (3,7-dimethyloct-6-enal); floralozone (3-(4-ethylphenyl)-2,2-dimethylpropanal/3-(2-ethylphenyl)-2,2-dimethylpropanal); vanillin (4-hydroxy-3-methoxybenzaldehyde); lilyall (3-(4-tert-butylphenyl)butanal); nerolidol (3,7,11-trimethyldodeca-1,6,10-trien-3-ol); carvacrol (2-trimethyl-5-propan-2-ylphenol); undecavertol ((E)-4-methyldec-3-en-5-ol); amber core (1-(2-tert-butylcyclohexyl)oxybutan-2-ol); benzyl alcohol (phenylmethanol); geraniol ((2E)-3,7-dimethylocta-2,6-dien-1-ol); linalool (3,7-dimethylocta-1,6-dien-3-ol); dimetol (2,6-dimethylheptan-2-ol), N- amyl methyl ketone (heptan-2-one); thibetolide (16-oxacyclohexadecan-1-one); Isoraldeine 95 (1-(2,6,6-trimethyl-1-cyclohex-2-enyl)pent-1-en-3-one); beta-ionone ((E)-4-(2,6,6-trimethyl-1-cyclohexenyl)but-3-en-2-one); gamma-octalactone (5-butyloxolan-2-one); alpha-damascone (1-(2,6,6-trimethyl-1-cyclohex-2-enyl)but-3-en-1-one); jasmonyl (mixture of 3-acetyloxynonyl acetate; nonane-1,3-diol); estragole ((1-methoxy-4-prop-2-enylbenzene); Rhubarb Furan (2,4-dimethyl-4-phenyloxolane); alpha- pinene (4,7,7-trimethylbicyclo(3.1.1)hept-3-ene); cresyl methyl ether (1-methoxy-4-methylbenzene). Odorant solutions are first prepared in DMSO and diluted to the final concentrations in DMEM. Information on the natural occurrence of odorants was obtained from the Good Scents Company Information System http://www.thegoodscentscompany.com/.

### Functional assay

Expression vectors for Ric-8B, Gαolf, and RTP1-S have been previously described (Von Dannecker et al., 2005, 2006). HEK293T cells are plated in 96-well microplates (0.3 × 10^5^ cells/well) and grown for 12–16 h. Cells are then transfected in the 96-well microplates with the expression vectors pcDNA3.1(-)Ric-8B (5 ng), pcDNA3.1(-)Gαolf (5 ng), pcDNA3.1(-)RTP1-S (10 ng) and one chosen pcDNA3.1(-)OR or pcDNA3.1(-)rho-OR (50 ng) using Lipofectamine™ 2000 (Invitrogen). Cells transfected with the pcDNA3.1(-) empty vector were used as negative control. Cells are also transfected with 10 ng of a plasmid containing the CRE-SEAP construction, where the expression of the secreted alkaline phosphatase (SEAP) is under regulation of the cAMP responsive elements, (pCRE-SEAP, Clontech) (Durocher et al., 2000; Malnic and Gonzalez-Kristeller, 2009). Wells containing cells transfected with a GFP reporter vector alone are included in each plate as a control for the potential difference in transfection efficiency. After transfection, cells are grown for additional 24 h. GFP expression is analyzed under the microscope to determine the transfection efficiency in each plate, which should be ≥70%.

Odorants are added to the plate wells (one different odorant per plate), which, is incubated for 24 h at 37°C and then at 65°C for 30 min to inactivate endogenous alkaline phosphatases and chilled on ice. OR activation by a specific odorant results in cAMP accumulation and activation of the CREB protein which in turn binds to the CRE sites to promote expression of the SEAP, which is secreted to the extracellular medium. To determine the amount of SEAP produced, 100 μL of the culture medium in each well is transferred to a new 96-well microplate (OptiPlate™-96 - PerkinElmer), 100 μL of a solution containing 1.2 mM fluorescent substrate 4-methylumbelliferyl phosphate (MUP, Sigma), 1 mM MgCl_2_, 10 mM L-homoarginine and 2M diethanolamine bicarbonate, pH 10.0 is added to each well and the reaction is incubated for 1 h at 37°C. Fluorescence is measured at 449 nm with a Victor3 V 1420 microplate reader (PerkinElmer). The results are expressed as arbitrary fluorescence units that reflect the activity of SEAP, according to the formula [F(OR + odorant)-F(empty pcDNA3.1 vector + odorant)]-[F(OR-odorant)-F(empty pcDNA3.1 vector - odorant)]. Only values of arbitrary fluorescence units greater than 3 × 10^5^ were considered. Statistical analysis was performed by using GraphPad Prism 5 software.

## Results

### Selection of human odorant receptors

**Figure 1:**
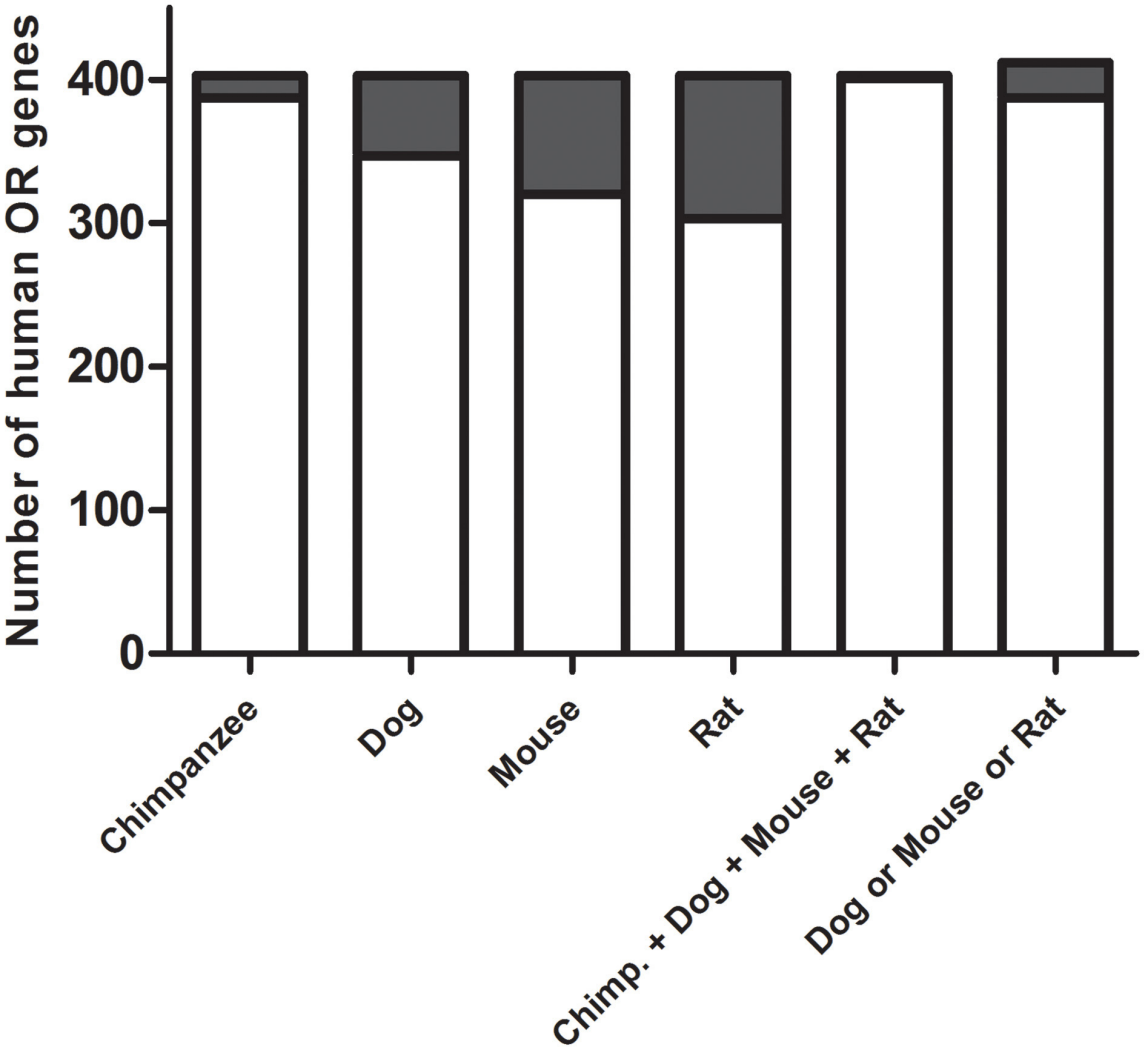
**Selection of the human odorant receptors**. The nucleotide sequences of the functional human ORs were aligned against the chimpanzee, dog, mouse and rat genomes. Odorant receptor sequences that were present in humans but not in the other species were selected. Gray regions of the bars correspond to the number of human ORs that show no similarity to the ORs in the other species. Only two odorant receptor sequences are present in the human genome but not in the other four species (chimp., dog, mouse and rat), and 25 odorant receptor sequences are present in the human and chimpanzee genomes but not in the dog, mouse, and rat genomes (dog and mouse and rat).

We first searched for human ORs that are present in the human genome but not in the mouse, rat, dog or chimpanzee genomes. In order to do this, 403 functional human OR DNA sequences were aligned against the chimpanzee, mouse, rat, and dog genome sequences. The selection of human ORs was based on the identity and coverage (percentage of sequence aligned) of the resulting alignments. As a cut-off, we established that the human OR sequences that show at least 70% of identity and 70% of coverage are present as a counterpart in the other species. Based on these criteria, we found that two human ORs are present only in humans (OR1C1 and OR2T10), while a total of 25 human ORs (OR11A1, OR13H1, OR14A16, OR14K1, OR14L1P, OR1C1, OR1G1, OR1L1, OR1L3, OR2B3, OR2G2, OR2M4, OR2M7, OR2T10, OR2T3, OR2T34, OR4K17, OR5AC2, OR5B17, OR5H14, OR5H15, OR5H6, OR2W5, OR7E125P, OR7E8P) are present in humans and chimpanzees, but not in the other species (Figure 1). OR2W5, OR7E125P and OR7E8P are pseudogenes (they contain frame shifts in their coding sequences) and were not considered in further analysis. Even though OR14L1P is annotated as a pseudogene (P), it contains an ORF of 293 amino acids, and contains all of the common OR motifs, therefore we did not considered it to be a pseudogene, even though it is a little bit shorter in its N-terminus when compared to the other ORs. Amino acid sequence identities among the 22 selected human OR genes ranges from 30.5 to 98.1% (see Supplementary Material 1).

### Functional analysis of the human ORs

We next used a high-throughput system (Malnic and Gonzalez-Kristeller, 2009) to search for agonists for a subset of the selected human ORs. The ORs OR1G1, OR1L3, OR2B3, OR2G2, OR2M4, OR2T10, OR2T34, OR5AC2, OR5B17 were randomly selected for functional analysis. These ORs show complete coding sequences which contain conserved motifs that are characteristic of the OR family (see Supplementary Material 2). They are all orphan ORs, except for OR1G1, which has been previously linked to odorants (Matarazzo et al., 2005; Sanz et al., 2005).

**Figure 2:**
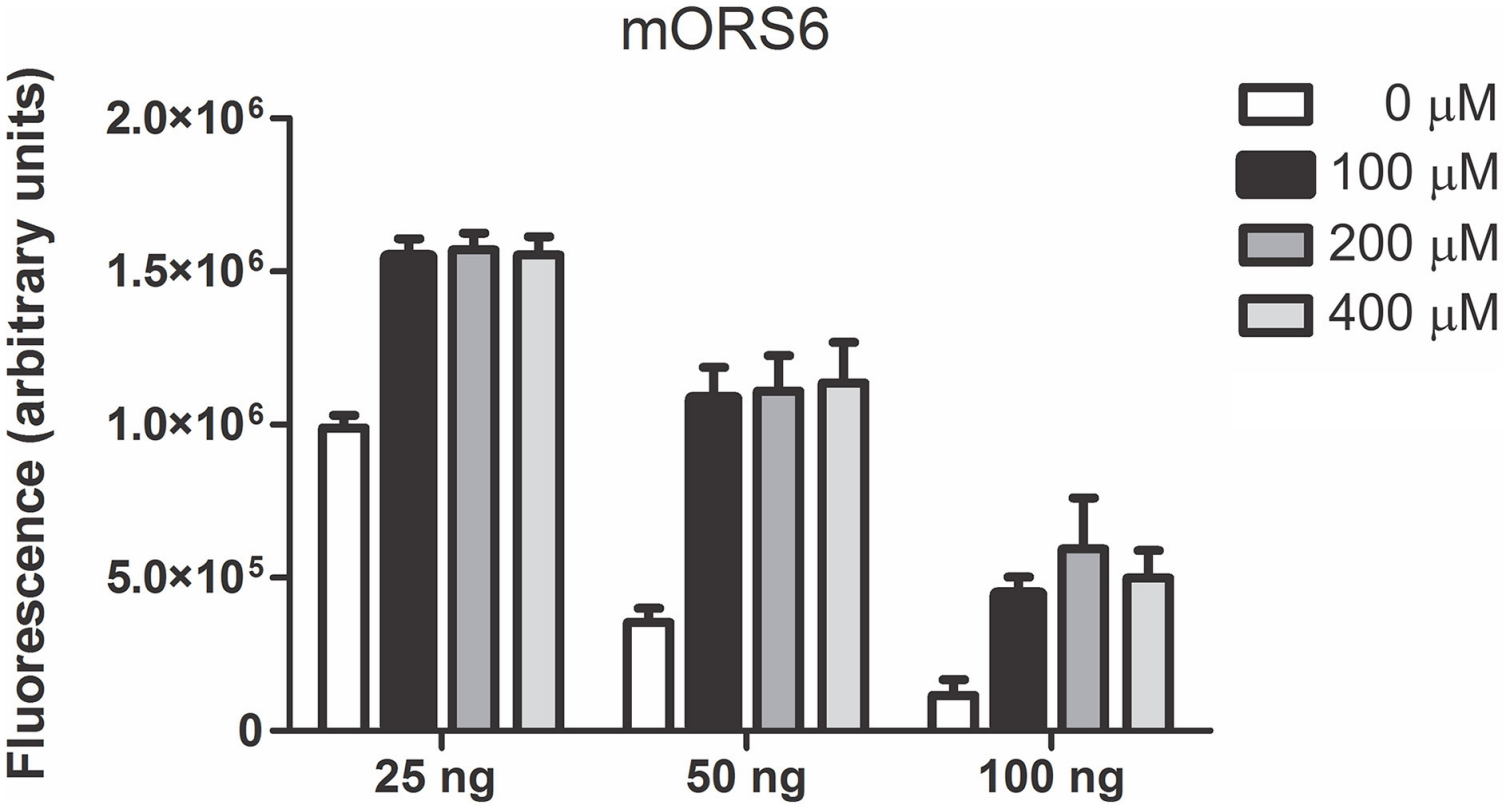
**Functional expression of mOR S6 in the heterologous screening system**. Responses of the mouse odorant receptor mOR S6 to nonanedioic acid were tested using the heterologous screening. Three different amounts of the mORS6 expression vector were used as indicated (25, 50, and 100 ng). Tested odorant concentrations were 100, 200, and 400 μM. SEAP activity is expressed in arbitrary fluorescence units.

The complete coding sequences from the ORs were amplified from human genomic DNA and cloned into the expression vector, with or without addition of the Rho tag to their N-terminus. HEK293T cells expressing Ric-8B, Gαolf, RTP1-S and a given OR, were stimulated with the odorants. As a positive control for the functional assay, the responses of the previously deorphanized mouse odorant receptor mOR S6 (Malnic et al., 1999) were analyzed. Cells expressing mOR S6 consistently responded to its agonist, nonanedioic acid (Figure 2). In these experiments we tested different amounts of the mOR S6 expression vector for the transfection of cells and observed that the use of increased amounts of the OR expression plasmid inhibits responses. It is possible that large amounts of OR expression are toxic to the cells, and that too little OR expression is not enough to distinguish between basal (no odorant) responses and odorant responses. We decided therefore to use 50 ng of the OR expressing plasmids in the next experiments.

**Figure 3:**
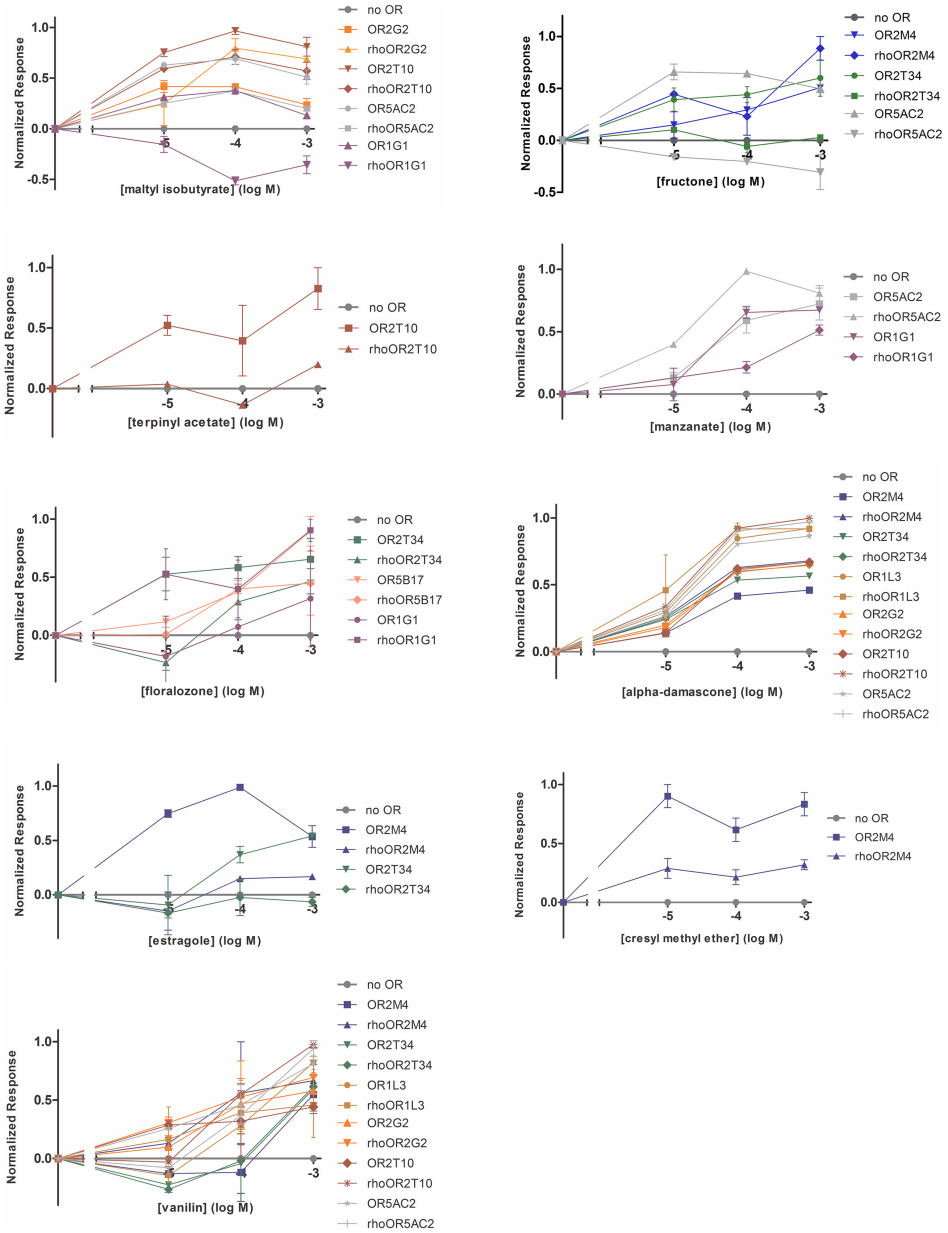
**Response profiles of the human ORs to odorants**. Dose response curves of the human ORs expressed in the heterologous expression system to the indicated odorants. SEAP activity was normalized as a percentage of the maximum response across a set of ORs. X-axis is the concentration of odorants in log Molar. Error bars represent s.e.m. over two replicates.

We screened the human ORs against a panel of 37 odorants with different chemical structures and odor types. Odorants were tested in three different concentrations (10, 100 or 1000 μM). Response profiles of the human ORs to some of the odorants are shown in Figure 3. Different ORs responded to different odorants, as expected. The complete odorant screening results are summarized in Table 1. Responses were obtained for all human ORs, but only five ORs produced a potent response to an odorant at the lowest (10 μM) odorant concentration (large black dots in Table 1, OR2M4, OR2T34, OR2T10, OR5AC2, and OR1G1). Out of the 37 odorants, 14 odorants were able to elicit a response, but only nine odorants elicited responses when applied at a 10 μM concentration (Table 1). The responses obtained for the untagged and Rho tagged versions of a given OR were highly similar for most of the ORs, but not always identical. In some cases, they were significantly different, like the responses to terpinyl acetate obtained for OR2T10 and the responses to fructone for OR5AC2 (Figure 3). It remains however to be determined whether both the untagged and rho tagged OR versions for each OR are expressed on the cell surface of the HEK293T cells.

**Table 1:**
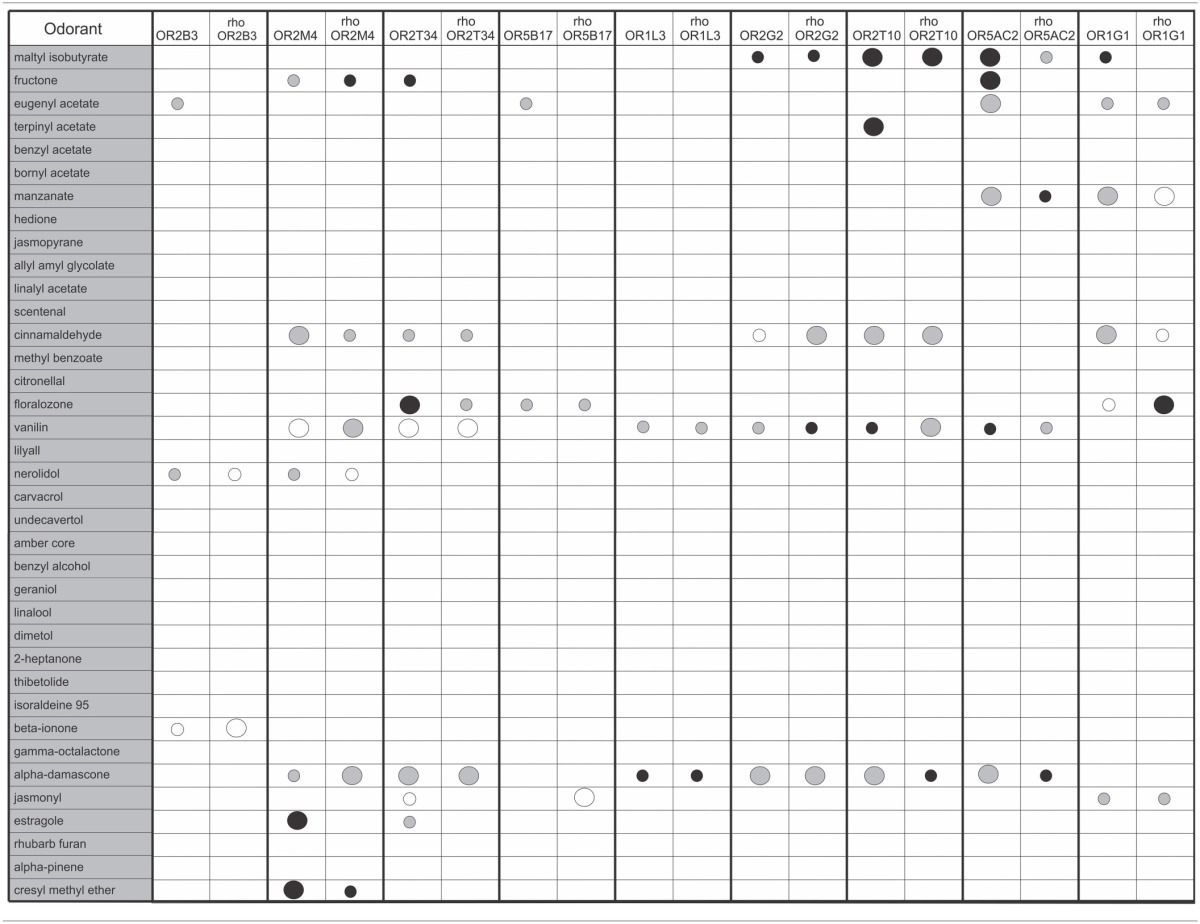
**Odorant responses of the human olfactory receptors**.

*The responses of the different human ORs (either with or without the Rho tag) to the selected odorants are shown. Responses are represented as dots with a shade of gray according to the odorant concentration at which the response was elicited: black (10 μM), gray (100 μM), and white (1000 μM). Small dots indicate values between 25 and 50% of the highest value found throughout the assay, while large dots indicate values higher than 50% of the highest value found throughout the assay*.

**Figure 4:**
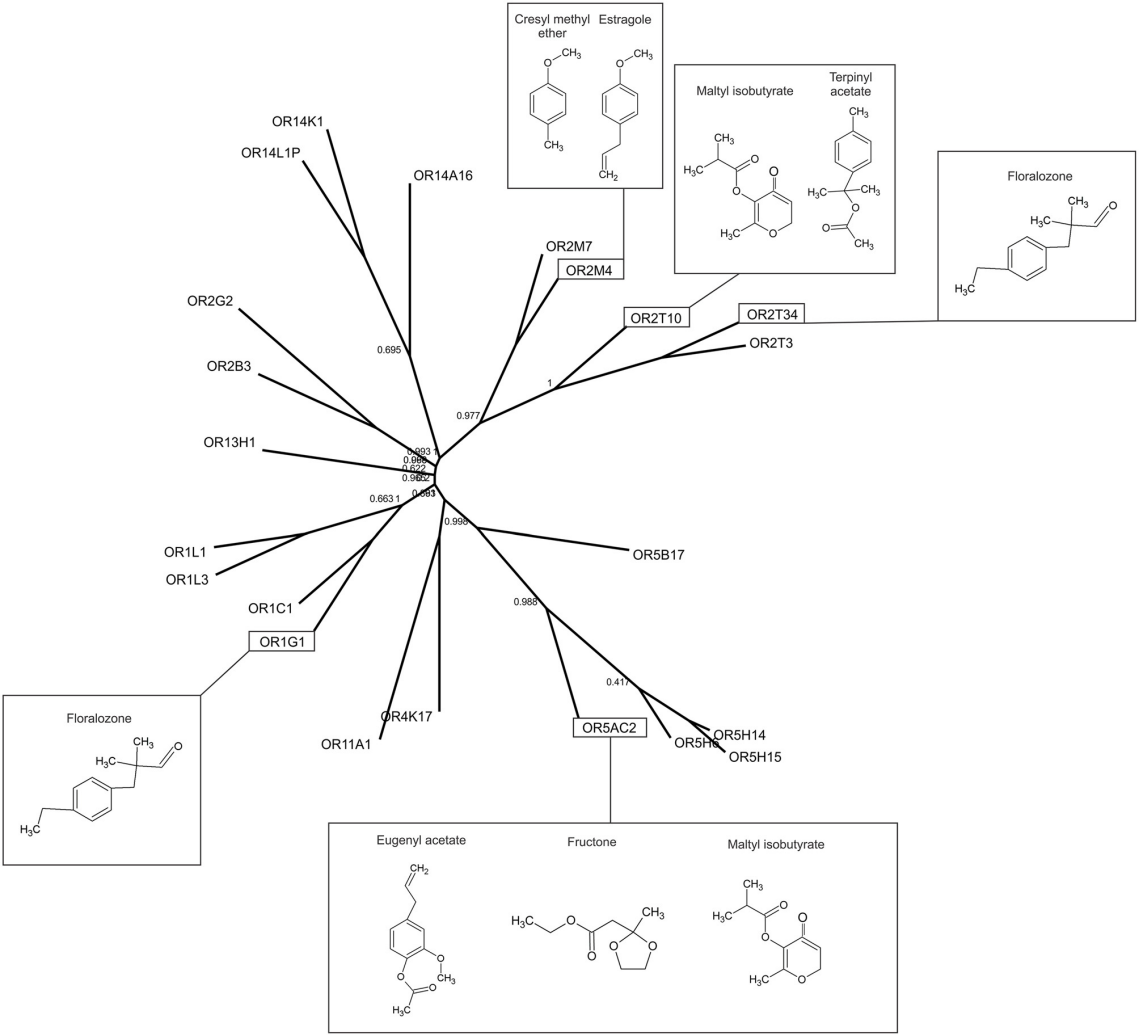
**Relationships among the selected human ORs**. Phylogenetic tree of the selected human OR amino acid sequences. The chemical structures of some of the agonists identified in this study are shown next to the corresponding responsive ORs.

The overall response profiles, shown in Table 1, are consistent with previous findings showing that odorant receptor coding is combinatorial and that while some ORs are narrowly tuned to a small number of odorants, others can be broadly tuned to a large number of odorants (Malnic et al., 1999; Saito et al., 2009; Nara et al., 2011). Also, while some of the odorants were able to activate a large fraction of the human ORs (like for example vanillin and α-damascone), other odorants activated only one or a few of the nine human ORs. For example, terpinyl acetate, which has an odor described as herbal, citrus, woody, was able to activate only OR2T10. The odorants estragole (a main constituent in basil oils), and cresyl methyl ether, an odorant naturally occurring in ylang ylang flower, potently activated only OR2M4. Interestingly, these odorants show highly related chemical structures (Figure 4), indicating that OR2M4 is tuned to odorants sharing common structural features present in these two odorants.

Several ORs responded preferentially to ester odorants, which have fruity odors. OR5AC2, for example, responded to the ester odorants maltyl isobutyrate, fructone, manzanate and eugenyl acetate, indicating that this receptor may be preferentially responsive to odorants containing ester functional groups (Figure 4). OR2T10 also responded to the ester odorants maltyl isobutyrate and terpinyl acetate. OR1G1, which was previously linked to the isoamyl acetate ester odorant (Matarazzo et al., 2005), also responded to ester odorants in this study. In addition, OR1G1 and OR2T34 responded to low concentrations of floralozone, an odorant with an ozone, marine and fresh odor. For the other ORs we only obtained responses at higher (100 or 1000 μM) odorant concentrations, suggesting either that they are low potency receptors or that their preferential agonists are not contained in the odorant panel we tested (Table 1, Supplementary Material 3).

## Discussion

Human ORs display a great diversity in their ligand preferences. Here we selected a specific group of human ORs and analyzed their odorant responses. We searched for ORs, which are present in humans but absent from other mammalian species. By using these criteria, we aimed to select receptors, which may be particularly relevant to the human species. Nine out of these human ORs were screened against a panel of 37 odorants. Since it is unfeasible to test the enormous number of odorants humans can discriminate (Bushdid et al., 2014), the selection of odorants to be used in the functional screening of ORs constitutes a very important step. Here we selected a panel of odorants humans are regularly confronted with, which are used as flavor and fragrance agents. The majority of the odorants in the panel can be found in nature. These odorants have odor types such as fruity, floral, herbal and citrus and must play important roles in food consumption.

High potency odorant responses (large black dots in Table 1) were obtained for five human ORs. Two human ORs responded to ester odorants with fruity odor types (OR2T10 and OR5AC2), two ORs responded to an odorant with a marine/ozone odor type (OR2T34 and OR1G1), and one receptor responded to two odorants with herbal and floral odor types (OR2M4). It is important to note that in our functional screening, both versions of the receptors (Rho tagged and untagged) were always analyzed in parallel. This strategy proved to be useful, since we noticed that for a few of the ORs, responses to some odorants differed depending on the absence or presence of the Rho tag. Since the human ORs are highly diverse in structure [their sequence identities range from 34 to 99% (Malnic et al., 2004)], it is possible that different receptors are differently affected by the presence of the Rho tag. That is, while for some receptors, it may enhance activation by a given odorant, for other ones it may hamper activation by a given odorant. For other ones, addition of the Rho tag may not affect odorant responses (for example, responses of OR2T10 to maltyl isobutyrate are identical between tagged and untagged receptor versions). In any case, additional studies should be performed in order to evaluate the effect of the Rho tag in these specific human ORs.

There are examples where nucleotide polymorphisms in odorant receptor genes are correlated with differences in odorant perception (Keller et al., 2007; Menashe et al., 2007; Mainland et al., 2014). The study of genetic influences on the perception of chemical stimulants should contribute to the understanding of the individual variation in food preferences (Dunkel et al., 2014). Particularly, these studies could explain individual preferences for herbs and spices. The smell of basil, for example, is not well characterized to date. Previous genetic studies indicated an association between the odor of basil and a bitter receptor gene (TAS2R60) (Knaapila et al., 2012), but no association to an odorant receptor was found. Here we show that the human OR2M4 is activated by the basil odorant estragole. No other human ORs had been linked to this odorant before. Future experiments should reveal whether additional ORs with related sequences are also able to recognize this odorant, and if individual genetic variations in OR2M4 influence the perception of basil.

How the different hit odorants, such as estragole, relate to the human specific ORs is an interesting question. Estragole is an odorant found in only a few foods (basil and parsley), why would it be recognized by evolutionarily selected ORs? The study of additional odorants in the functional screening should help to clarify this question. Interestingly, recent studies identified a limited set of about 230 foodborne volatiles, which largely represent the aroma-relevant odor space of most human foods, and suggest that these key food odorants are more likely to result in cognate odorant/receptor pairs in functional screening assays as compared to non-key food odorants (Dunkel et al., 2014). These studies should contribute to the design of appropriate odorant panels to be used in the functional screening of human ORs. In the case of our selected human ORs, future experiments should include odorants which are evolutionary selected by nature, not only key food odorants, but also body odors, which are for example involved in offspring identification or other types of olfactory communication (Dunkel et al., 2014).

## Conclusions

To identify biologically relevant human ORs we selected ORs present in the human genome but absent from the genomes of other mammalian species. An initial functional screening identified odorants that activate these previously orphan human ORs. A more systematic approach, where physico chemical features of the hit odorants, such as C-atom chain length or functional groups, are changed, should help to better characterize the odorant/receptor pairs identified in this study.

## Author contributions

DG and JN carried out the molecular biology and functional screening experiments. PG performed the bioinformatics analysis. BM and DG participated in the design of the study. BM wrote the manuscript. All authors read and approved the final manuscript.

### Conflict of interest statement

The authors declare that the research was conducted in the absence of any commercial or financial relationships that could be construed as a potential conflict of interest.
