# Supplementary material for: Identification of agonists for a group of human odorant receptors
Source: Front Pharmacol. 2015 Mar 3;6:35. doi: 10.3389/fphar.2015.00035 (PMC4347425; doi:10.3389/fphar.2015.00035)

***Supplementary Material***

# Identification of agonists for a group of human odorant receptors

# Daniela Gonzalez-Kristeller^1^, João Batista Plácido do Nascimento^1^, Pedro AF Galante^2^ and Bettina Malnic^1^

1Institute of Chemistry, Department of Biochemistry, University of São Paulo, São Paulo, SP, Brazil

^2^Centro de Oncologia Molecular, Hospital Sírio-Libanês – São Paulo, SP, Brazil.

*** Correspondence:** Bettina Malnic, ^1^Department of Biochemistry, University of São Paulo, Av. Prof. Lineu Prestes, 748, CEP 05508-000, São Paulo, SP, Brazil [bmalnic@iq.usp.br](mailto:bmalnic@iq.usp.br)

**Supplementary material 1- Percent identity matrix.** Amino acid sequence identities among all 22 human ORs were obtained using ClustalW2 multiple sequence alignment tool (http://www.ebi.ac.uk).

Percent Identity Matrix - created by Clustal2.1

1: OR1C1 100.00 52.40 50.32 50.80 40.97 40.89 41.42 41.37 40.06 40.51 37.70 38.02 40.71 39.55 38.46 40.84 38.85 39.29 38.66 41.04 40.06 39.59

2: OR1G1 52.40 100.00 52.58 49.36 44.84 42.81 44.01 41.37 38.91 38.71 38.34 38.34 40.38 40.84 42.31 42.44 37.06 37.34 37.50 41.18 38.26 40.27

3: OR1L1 50.32 52.58 100.00 78.06 41.75 41.61 42.86 43.14 45.13 41.56 39.35 39.35 43.23 38.39 37.10 44.19 39.68 41.04 39.81 38.94 35.39 41.44

4: OR1L3 50.80 49.36 78.06 100.00 38.51 38.46 39.29 41.50 43.13 39.10 39.42 38.78 42.77 40.32 38.59 44.84 38.98 43.32 37.50 37.99 35.78 38.70

5: OR5H14 40.97 44.84 41.75 38.51 100.00 85.81 86.41 60.91 42.86 38.11 34.84 34.84 37.10 38.71 37.74 37.10 33.87 37.34 34.30 36.30 30.52 33.79

6: OR5H15 40.89 42.81 41.61 38.46 85.81 100.00 81.23 59.28 42.12 38.71 33.55 33.23 34.94 37.94 37.82 36.66 34.82 34.42 35.26 36.27 30.55 32.76

7: OR5H6 41.42 44.01 42.86 39.29 86.41 81.23 100.00 59.87 41.37 38.51 36.62 36.62 37.86 39.48 38.19 35.92 33.97 36.69 35.81 37.62 31.92 34.13

8: OR5AC2 41.37 41.37 43.14 41.50 60.91 59.28 59.87 100.00 43.28 36.60 36.25 36.25 36.16 38.76 36.81 39.74 37.86 40.85 39.94 37.29 31.80 35.84

9: OR5B17 40.06 38.91 45.13 43.13 42.86 42.12 41.37 43.28 100.00 40.65 37.62 37.30 37.74 38.51 37.10 39.16 36.22 39.87 36.33 38.83 34.39 36.18

10: OR4K17 40.51 38.71 41.56 39.10 38.11 38.71 38.51 36.60 40.65 100.00 33.02 32.70 37.86 36.04 35.92 37.99 35.99 35.41 36.54 38.03 35.48 36.55

11: OR2T3 37.70 38.34 39.35 39.42 34.84 33.55 36.62 36.25 37.62 33.02 100.00 98.11 60.58 48.23 45.83 37.94 33.86 39.29 35.03 33.33 34.08 37.54

12: OR2T34 38.02 38.34 39.35 38.78 34.84 33.23 36.62 36.25 37.30 32.70 98.11 100.00 59.94 47.91 45.51 37.94 33.86 39.61 35.35 33.01 33.76 37.54

13: OR2T10 40.71 40.38 43.23 42.77 37.10 34.94 37.86 36.16 37.74 37.86 60.58 59.94 100.00 51.77 50.00 42.12 37.18 42.21 33.76 37.05 36.13 38.57

14: OR2M4 39.55 40.84 38.39 40.32 38.71 37.94 39.48 38.76 38.51 36.04 48.23 47.91 51.77 100.00 73.31 41.16 38.26 41.88 36.45 40.13 35.28 35.49

15: OR2M7 38.46 42.31 37.10 38.59 37.74 37.82 38.19 36.81 37.10 35.92 45.83 45.51 50.00 73.31 100.00 41.48 37.82 41.23 36.33 38.69 35.81 36.86

16: OR2B3 40.84 42.44 44.19 44.84 37.10 36.66 35.92 39.74 39.16 37.99 37.94 37.94 42.12 41.16 41.48 100.00 55.31 42.86 37.10 39.14 34.63 38.91

17: OR2G2 38.85 37.06 39.68 38.98 33.87 34.82 33.97 37.86 36.22 35.99 33.86 33.86 37.18 38.26 37.82 55.31 100.00 44.16 39.05 32.25 35.58 36.18

18: OR13H1 39.29 37.34 41.04 43.32 37.34 34.42 36.69 40.85 39.87 35.41 39.29 39.61 42.21 41.88 41.23 42.86 44.16 100.00 37.46 35.10 33.99 38.01

19: OR11A1 38.66 37.50 39.81 37.50 34.30 35.26 35.81 39.94 36.33 36.54 35.03 35.35 33.76 36.45 36.33 37.10 39.05 37.46 100.00 34.64 33.44 37.33

20: OR14A16 41.04 41.18 38.94 37.99 36.30 36.27 37.62 37.29 38.83 38.03 33.33 33.01 37.05 40.13 38.69 39.14 32.25 35.10 34.64 100.00 50.81 51.89

21: OR14K1 40.06 38.26 35.39 35.78 30.52 30.55 31.92 31.80 34.39 35.48 34.08 33.76 36.13 35.28 35.81 34.63 35.58 33.99 33.44 50.81 100.00 48.46

22: OR14L1P 39.59 40.27 41.44 38.70 33.79 32.76 34.13 35.84 36.18 36.55 37.54 37.54 38.57 35.49 36.86 38.91 36.18 38.01 37.33 51.89 48.46 100.00

**Supplementary Material 2- Amino acid sequence alignment of the selected human ORs.** Amino acid sequences were aligned using multiple sequence alignment by ClustalW (<http://www.genome.jp/tools/clustalw/>). Regions of conserved motifs that are characteristic of the OR family are boxed in red.

HOR2B3 -----MNWENESSPKEFILLGFSDRAWLQMPLFVVLLISYTITIFGNVSIMMVCILDPKL

HOR2G2 --MGMVRHTNESNLAGFILLGFSDYPQLQKVLFVLILILYLLTILGNTTIILVSRLEPKL

HOR1G1 -----MEGKNLTSISECFLLGFSEQLEEQKPLFGSFLFMYLVTVAGNLLIILVIITDTQL

HOR1L3 -----MGMSNLTRLSEFILLGLSSRSEDQRPLFALFLIIYLVTLMGNLLIILAIHSDPRL

HOR5AC2 ---MDISEGNKTLVTEFVLTGLTDRPWLHVLFFVVFLVVYLITMVGNLGLIVLIWNDPHL

HOR5B17 -------MENNTEVSEFILLGLTNAPELQVPLFIMFTLIYLITLTGNLGMIILILLDSHL

HOR2T10 -----MRLANQTLGGDFFLLGIFSQISHPGRLCLLIFSIFLMAVSWNITLILLIHIDSSL

HOR2T34 MCSGNQTSQNQTASTDFTLTGLFAESKHAALLYTVTFLLFLMALTGNALLILLIHSEPRL

HOR2M4 -----MVWENQTFNSIFILLGIFNHSPTHTFLFSLVLGIFSLALMENISMVLLIYIEKQL

* : * *: : : ::: * ::: : *

HOR2B3 HTPMYFFLTNLSILDLCYTTTTVPHMLVNIGCNKKTISYAGCVAHLIIFLALGATECLLL

HOR2G2 HMPMYFFLSHLSFLYRCFTSSVIPQLLVNLWEPMKTIAYGGCLVHLYNSHALGSTECVLP

HOR1G1 HTPMYFFLANLSLADACFVSTTVPKMLANIQIQSQAISYSGCLLQLYFFMLFVMLEAFLL

HOR1L3 QNPMYFFLSILSFADICYTTVIVPKMLVNFLSEKKTISYAECLAQMYFFLVFGNIDSYLL

HOR5AC2 HMPMYLFLGGLAFSDACTSTSITPRMLVNFLDKTAMISLAECITQFYFFASSATTECFLL

HOR5B17 HTPMYFFLSNLSLAGIGYSSAVTPKVLTGLLIEDKAISYSACAAQMFFCAVFATVENYLL

HOR2T10 HTPMYFFINQLSLIDLTYISVTVPKMLVNQLAKDKTISVLGCGTQMYFYLQLGGAECCLL

HOR2T34 HTPMYFFISQLALMDLMYLCVTVPKMLVGQVTGDDTISPSGCGIQMFFHLTLAGAEVFLL

HOR2M4 HTPMYFLLSQLSLMDLMLICTTLPKMIFSYLSGKKSISLAGCGTQIFFYVSLLGAECFLL

: ***::: *:: *::: . *: * :: : *

HOR2B3 AVMSFDRYVAVCRPLHYVVIMNYWFCLRMAAFSWLIGFGNSVLQSSLTLNMPRCGHQEVD

HOR2G2 AVMSCDRYVAVCRPLHYTVLMHIHLCMALASMAWLSGIATTLVQSTLTLQLPFCGHRQVD

HOR1G1 AVMAYDCYVAICHPLHYILIMSPGLCIFLVSASWIMNALHSLLHTLLMNSLSFCANHEIP

HOR1L3 AAMAINRCVAICNPFHYVTVMNRRCCVLLLAFPITFSYFHSLLHVLLVNRLTFCTSNVIH

HOR5AC2 VMMAYDRYVAICNPLLYPVMMSNKLSAQLLSISYVIGFLHPLVHVSLLLRLTFCRFNIIH

HOR5B17 SSMAYDRYAAVCNPLHYTTTMTTRVCACLAIGCYVIGFLNASIQIGDTFRLSFCMSNVIH

HOR2T10 AAMAYDRYVAICHPLRYSVLMSHRVCLLLASGCWFVGSVDGFMLTPIAMSFPFCRSHEIQ

HOR2T34 AAMAYDRYAAVCRPLHYPLLMNQRVCQLLVSACWVLGMVDGLLLTPITMSFPFCQSRKIL

HOR2M4 AVMAYDRYVAICHPLQYTILMNPKLCVFMTVASWTLGSLDGIIVLAAVLSFSYCSSLEIH

*: : .*:*.*: * * . : . : :. * :

HOR2B3 HFFCEVPALLKLSCADTKPIEAELFFFSVLILLIPVTLILISYGFIAQAVLKIRSAEGRQ

HOR2G2 HFICEVPVLIKLACVGTTFNEAELFVASILFLIVPVSFILVSSGYIAHAVLRIKSATRRQ

HOR1G1 HFFCDINPLLSLSCTDPFTNELVIFITGGLTGLICVLCLIISYTNVFSTILKIPSAQGKR

HOR1L3 HFFCDVNPVLKLSCSSTFVNEIVAMTEGLASVMAPFVCIIISYLRILIAVLKIPSAAGKH

HOR5AC2 YFYCEILQLFKISCNGPSINALMIFIFGAFIQIPTLMTIIISYTRVLFDILKKKSEKGRS

HOR5B17 HFFCDKPAVITLTCSEKHISELILVLISSFNVFFALLVTLISYLFILITILKRHTGKGYQ

HOR2T10 HFFCEVPAVLKLSCSDTSLYKIFMYLCCVIMLLIPVTVISVSYYYIILTIHKMNSVEGRK

HOR2T34 SFFCETPALLKLSCSDVSLYKMLTYLCCILMLLTPIMVISSSYTLILHLIHRMNSAAGRR

HOR2M4 HFFCDVAALLPLSCTETSAFERLLVICCVVMLIFPVSVIILSYSHVLRAVIHMGSGESRR

* *: :: ::* : . * : : : :

HOR2B3 KAFGTCGSHMIVVSLFYGTAIYMYLQPPSSTSKDWGKMVSLFYGIITSMLNSLIYSLRNK

HOR2G2 KAFGTCFSHLTVVTIFYGTIIFMYLQPAKSRSRDQGKFVSLFYTVVTRMLNPLIYTLRIK

HOR1G1 KAFSTCSSHLSVVSLFFGTSFCVDFSSPSTHSAQKDTVASVMYTVVTPMLNPFIYSLRNQ

HOR1L3 KAFSTCSSHLTVVILFYG-SISYVYLQPLSSYTVKDRIATINYTVLTSVLNPFIYSLRNK

HOR5AC2 KAFSTCGAHLLSVSLYYGTLIFMYVRPASGLAEDQDKVYSLFYTIIIPLLNPFIYSLRNK

HOR5B17 KPLSTCGSHLIAIFLFYITVIIMYIRPSSSHSMDTDKIASVFYTMIIPMLSPIVYTLRNK

HOR2T10 KAFTTCSSHITVVSLFYGAAIYNYMLPSSYQTPEKDMMSSFFYTILTPVLNPIIYSFRNK

HOR2T34 KALATCSSHMIIVLLLFGASFYTYMLRSSYHTAEQDMMVSAFYTIFTPVLNPLIYSLRNK

HOR2M4 KAFTTCSSHLSVVGLYYGAAMFMYMRPASKHTPDQDKMVSAFYTILTPMLNPLIYSLRNK

*.: ** :*: : : : : . . . : * :. :*..::*::* :

HOR2B3 DMKEAFKRLMPRIFFCX-------------

HOR2G2 EVKGALKKVLAKALGVNIL-----------

HOR1G1 EIKSSLRKLIWVRKIHSP------------

HOR1L3 DMKRGLQKLINKIKSQMSRFSTKTNKICGP

HOR5AC2 KVMHALRRVIRK------------------

HOR5B17 DVKNAFMKVVEKAKYSLDSVF---------

HOR2T10 DVTRALKKMLSVQKPPY-------------

HOR2T34 DVTRALRSMMQSRMNQEK------------

HOR2M4 EVFRALQKVLKKRKLI--------------

.: .: ::

**Supplementary material 3- Response profiles of human ORs to odorants.** Dose response curves of the human ORs expressed in the heterologous expression system to the indicated odorants. SEAP activity was normalized as a percentage of the maximum response across a set of ORs. X-axis is the concentration of odorants in log Molar. Error bars represent s.e.m. over two replicates.


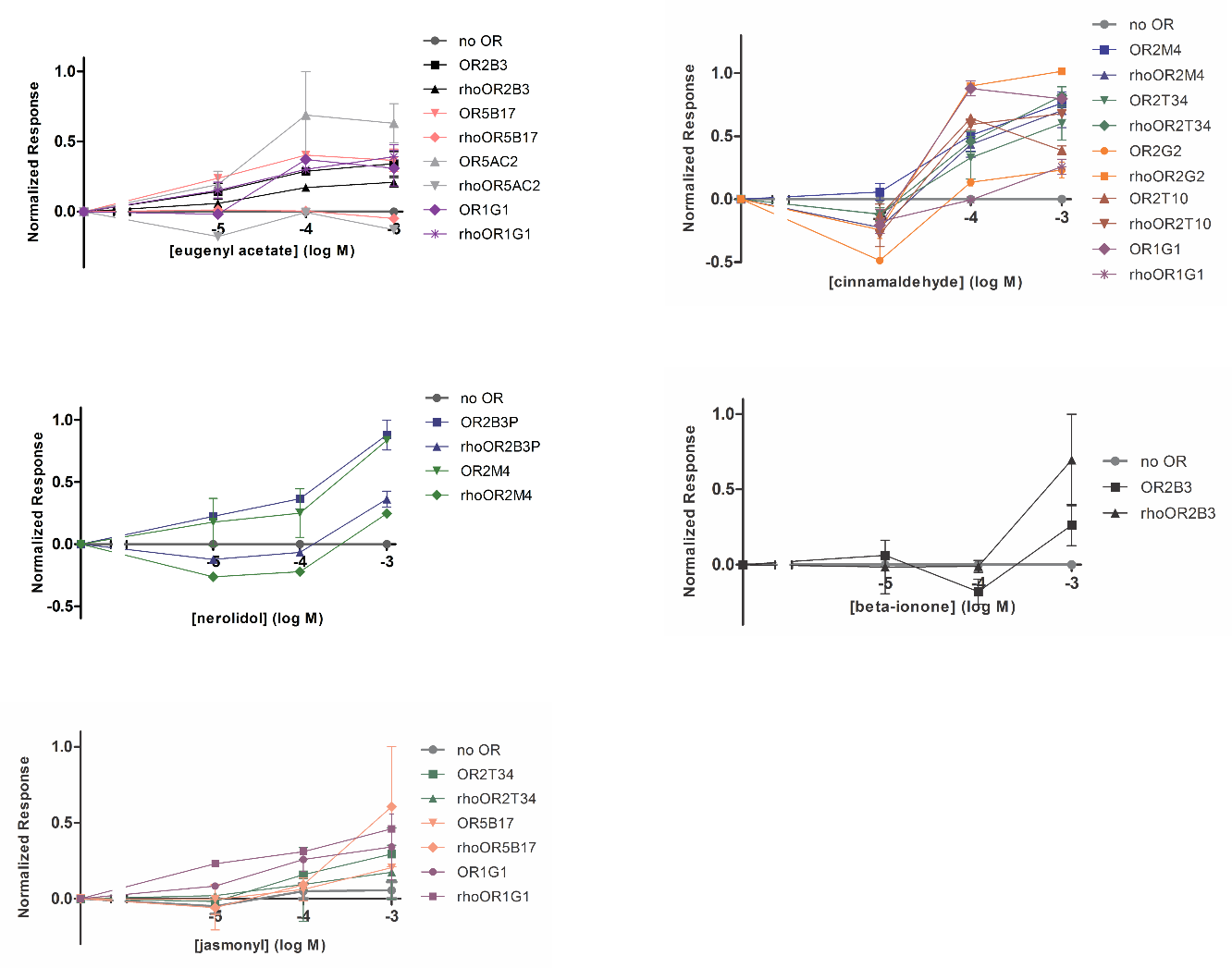

Supplement: Supplementary file 1 [file DataSheet1.DOCX]
